# Dexmedetomidine Reduces Incidences of Ventricular Arrhythmias in Adult Patients: A Meta-Analysis

**DOI:** 10.1155/2022/5158362

**Published:** 2022-06-01

**Authors:** Qiaonan Zhong, Ashish Kumar, Abhishek Deshmukh, Courtney Bennett

**Affiliations:** ^1^Department of Medicine, Mayo Clinic, Rochester, NY, USA; ^2^Department of Medicine, Department of Internal Medicine, Cleveland Clinic Akron General Hospital, Akron, OH, USA; ^3^Department of Cardiovascular Medicine, Mayo Clinic, Rochester, NY, USA

## Abstract

**Purpose:**

To assess the antiarrhythmic properties of dexmedetomidine in patients in the intensive care unit.

**Methods:**

A literature review was conducted with Ovid MEDLINE (R), Cochrane Central Register of Controlled Trials, Cochrane Database of Systematic Reviews, Embase, and Scopus. *Study Selection*. Randomized controlled trials were included, examining the incidence of ventricular arrhythmias, ventricular tachycardia, or ventricular fibrillation with dexmedetomidine compared to placebo or an alternative sedative agent. For each publication that met the selection criteria, the patient demographics, incidence of arrhythmias, mortality, and adverse events were collected. Data extraction was carried out by two authors independently.

**Results:**

We identified 6 out of 126 studies that met the selection criteria for our meta-analysis, all of which focused on the perioperative cardiac surgery period. Patients receiving dexmedetomidine demonstrated a significant reduction of the overall incidence of ventricular arrhythmias (RR 0.35, 95% CI 0.16, 0.76). In particular, dexmedetomidine significantly decreased the risk of ventricular tachycardia compared with control (RR 0.25, 95% CI 0.08, 0.80, I^2^ 0%). Regarding adverse events, dexmedetomidine significantly increased the frequency of bradycardia (RR 2.78 95% CI 2.00, 3.87). However, there was no significant difference in mortality (RR 0.59 95% CI 0.12, 3.02).

**Conclusion:**

From this meta-analysis, we report a decreased incidence of ventricular tachycardia with dexmedetomidine in critically ill patients. This result favors the use of dexmedetomidine for its antiarrhythmic properties.

## 1. Introduction

Ventricular arrhythmias (VA), including ventricular tachycardia (VT) and ventricular fibrillation (VF), are associated with high mortality and morbidity resulting in prolonged hospitalizations [1–4]. Defibrillation/cardioversion, antiarrhythmic drug therapy, and sedation are first-line approaches to VA and electrical storm management [5]. Most ICU sedatives, however, have significant side effect profiles. Benzodiazepines are associated with respiratory and cardiovascular depression, delirium, propylene glycol-related kidney injury, and drug interactions [6–8]. While providing efficacious sedation, propofol provides no analgesic effect and requires an invasive airway for continuous infusion. Moreover, it is associated with hypotension, bradycardia, arrhythmias, neuroexcitatory effects (seizure-like activity and myoclonus), respiratory acidosis, pancreatitis, hypertriglyceridemia, and propofol-related infusion syndrome [6, 9].

Dexmedetomidine is an alpha-2 presynaptic receptor agonist with sedative, analgesic, and anti-inflammatory properties [10, 11]. Recently, dexmedetomidine has been suggested as an alternative agent for sedation in VT due to its antiarrhythmic properties through decreased catecholamine release, prolonged refractory period, and increased vagal tone [12]. In addition, dexmedetomidine is a highly selective agonist that does not interact with the gamma-aminobutyric acid (GABA) receptors. Thus, its analgesic properties are opioid-sparing, which is unique among traditional ICU sedatives and avoids the issue of respiratory depression with over-sedation [13–15]. Moreover, current studies suggest dexmedetomidine may reduce duration of ventilation [16, 17] and ICU length of stay [18], delirium [19, 20], atrial fibrillation [11], renal injury [21], and myocardial ischemia [22]. Several randomized controlled trials (RCTs) have evaluated the role of dexmedetomidine in VA, but these studies are limited by sample size [20, 22–27]. The use of dexmedetomidine may result in the potential side effects of bradycardia and hypotension [28–31]. Herein, we conducted a meta-analysis to examine the effect of dexmedetomidine on the VA occurrence and potential adverse effects.

## 2. Materials and Methods

### 2.1. Data Sources and Search Strategies

The present meta-analysis and systematic review was performed in accordance with the PRISMA (Preferred Reporting Items for Systematic Reviews and Meta-Analyses) guidelines. Two authors independently carried out the literature search of several databases from inception to November 25, 2020, limited to the English language and excluding animal studies. The databases included Ovid MEDLINE (R) Epub Ahead of Print, In-Process & Other Non-Indexed Citations and Daily, EBM Reviews—Cochrane Central Register of Controlled Trials, EBM Reviews—Cochrane Database of Systematic Reviews, Embase, and Scopus.

The search strategy was designed and conducted by an experienced librarian with input from the study's investigators. Controlled vocabulary supplemented with keywords was used to search for randomized controlled studies and meta-analyses describing the association of dexmedetomidine with ventricular arrhythmias. The detailed search strategy is available in Appendix 1. Additional studies were identified from the review of published prior meta-analyses with the above search strategy. All eligible studies met the following inclusion criteria: (1) organized as a randomized controlled trial (RCT); (2) compared dexmedetomidine with placebo or alternate sedative in adult ICU patients; (3) reported primary or secondary outcomes of ventricular arrhythmia (VA), defined as VT or VF; (4) patient population age ≥18 years of age. Studies were excluded if they (1) did not report frequency of VA; (2) were not in English.

### 2.2. Data Extraction

The following information was extracted for each included publication: author, publication year, number of patients, sedation goal, dexmedetomidine dose and duration, comparison agent (placebo or alternative sedation) (Table 1). Baseline patient characteristics were tabulated when provided (Table 2). The following outcomes were also collected: incidence of ventricular tachycardia, ventricular fibrillation, atrial fibrillation, duration of mechanical ventilation, length of ICU stay, length of hospitalization, mortality, bradycardic events, and hypotensive events. Data extraction was carried out by two authors independently, and any disparity was solved by mutual consensus after consultation with all authors.

### 2.3. Quality Assessment

We used the Cochrane risk of bias version 2 (RoB2) form to account for biases associated with each trial. The RoB form contains sets of questions specific to a particular domain. The risk of bias in each of these domains was stratified into “low risk,” “high risk,” or “some concerns” based on answers to the specific questions. An overall bias was computed from these subscores. Risk of bias assessment of included studies was performed using two authors independently. Any disparity was resolved by mutual consensus.

### 2.4. Statistical Analysis

In the present analysis, we used the random effect, Mantel–Haenszel method with Paule–Mandel (PM) estimator of tau^2^ and Hartung–Knapp–Sidik–Jonkman adjustment to calculate risk ratio (RR) with 95% confidence interval (CI). The Q-profile method was used to compute the confidence interval of tau^2^ and tau. A continuity correction of 0.1 was used in studies with zero cell frequencies. I^2^ statistic was used to assess the heterogeneity between studies. A funnel plot was used to evaluate publication bias. All statistical analysis was carried out using R version 4.0.3 metapackage.

## 3. Results

### 3.1. Included Studies

**Figure 1:**
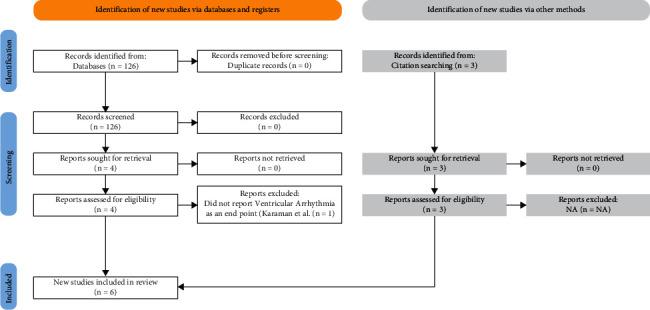
Study flow diagram.

A total of 126 records were identified in our search of databases. Three additional articles were identified from the review of published meta-analyses. After reviewing the abstracts in accordance with our inclusion criteria, the full text of 7 articles was retrieved and assessed for eligibility. One article did not meet our exclusion criteria; the remaining 6 were included in our analysis (Figure 1).

### 3.2. Basic Study Characteristics

With the six randomized control trials included in this meta-analysis, the total number of patients was 1001, with the number of patients ranging between 76 and 290 for each study (Table 1). Three studies compared dexmedetomidine with placebo [22, 25, 27]. Two studies compared dexmedetomidine with propofol [23, 24]. The remaining study compared dexmedetomidine with morphine [20].

### 3.3. Outcomes

**Figure 2:**
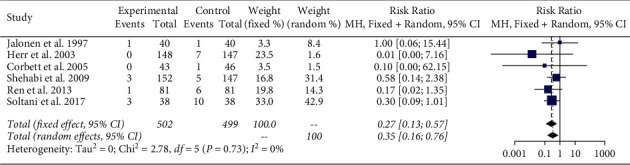
Meta-analysis of ventricular arrhythmias (VA).

**Figure 3:**
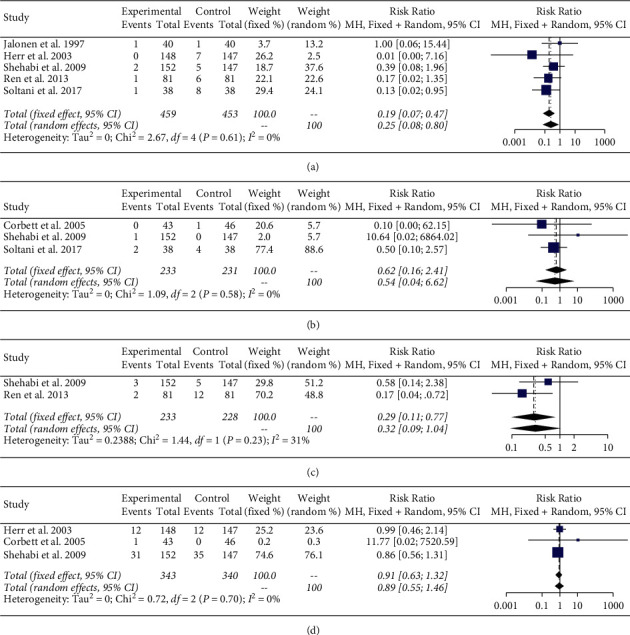
Meta-analysis of (a) ventricular tachycardia, (b) ventricular fibrillation, (c) supraventricular tachycardia, and (d) atrial fibrillation.

The meta-analysis of the included six studies demonstrates a significant reduction in the risk of ventricular arrhythmias in patients receiving dexmedetomidine (RR 0.35, 95% CI 0.16, 0.76, I^2^ 0%) compared with control (Figure 2). Furthermore, the analysis demonstrated that dexmedetomidine significantly decreased the risk of VT (RR 0.25, 95% CI 0.08, 0.80, I^2^ 0%) compared with control (Figure 3(a)). There was no significant heterogeneity amongst these studies. There was no difference in the risk of VF in patients receiving dexmedetomidine (RR 0.54 95% CI 0.04, 6.62, I^2^ 0%) compared with control (Figure 3(b)). Additionally, there was no significant difference in the incidence of supraventricular tachycardia (RR 0.32 95% CI 0.09, 1.07, I^2^ 31%) (Figure 3(c)) or atrial fibrillation (RR 0.89 95% CI 0.55, 1.46, I^2^ 0%) (Figure 3(d)).

**Figure 4:**
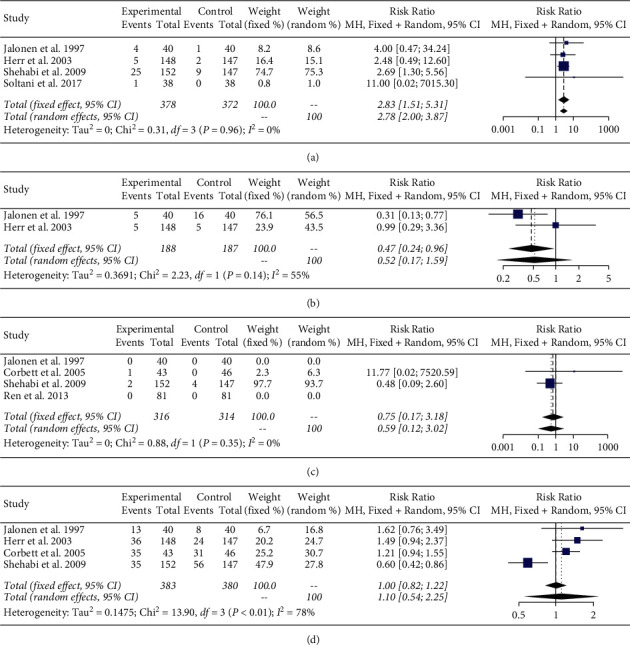
Meta-analysis of (a) bradycardia, (b) tachycardia, (c) mortality, and (d) hypotension.

Dexmedetomidine was found to significantly increase the risk of bradycardia (RR 2.78 95% CI 2.00, 3.87, I^2^ 0%) compared with control (Figure 4(a)). There was no significant difference in the risk of tachycardia (RR 0.52 95% CI 0.17, 1.59, I^2^ 55%) (Figure 4(b)), mortality (RR 0.59 95% CI 0.12, 3.02, I^2^ 0%) (Figure 4(c)), or hypotension (RR 1.10 95% CI 0.54, 2.25, I^2^ 71%) (Figure 4(d)) with dexmedetomidine compared with control.

### 3.4. Risk of Bias and Sensitivity Analyses

**Figure 5:**
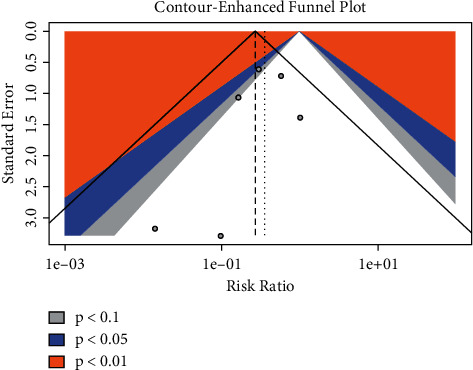
Funnel plot for publication bias of ventricular arrhythmias.

**Figure 6:**
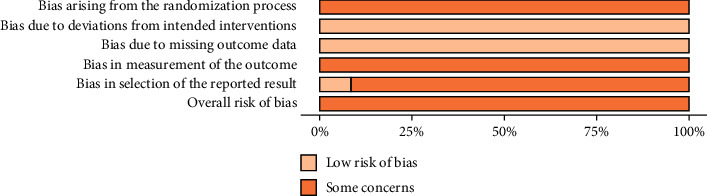
Risk of bias of included studies.

The funnel plot (Figure 5) suggests asymmetry in the distribution of studies in the right lower triangle which falls in the nonsignificant portion of the counterenhanced funnel plot, indicating the possibility of publication bias for ventricular arrhythmia. We refrained from using statistical tests for the interpretation of publication bias considering the small number of included studies, and the statistical tests are underpowered under such circumstances. Assessment of the study quality revealed some concerns associated with all included studies, according to the Cochrane ROB2 form (Figure 6).

## 4. Discussion

### 4.1. Cardiac Arrhythmia

Sustained VT and VF are the most common arrhythmic causes of sudden cardiac death [32]. Medical treatment is limited to a handful of antiarrhythmic drugs, the most common being amiodarone [1, 33]. The management of ventricular arrhythmias and electrical storm also incorporates medical and surgical interruption of the sympathetic nervous system, including sedative agents in the former. Dexmedetomidine has become a popular sedative agent in ICU patients. However, the antiarrhythmic mechanisms of action of dexmedetomidine have not been elucidated.

The classical hypothesis is that dexmedetomidine increases vagal activity by stimulating alpha-2 adrenoreceptors found on the nucleus ambiguous and the dorsal motor nucleus of the vagus [13, 34–37]. These centers of parasympathetic activity upregulate acetylcholine to bind with G protein-coupled muscarinic acetylcholine receptors in cardiac myocytes. Interaction of acetylcholine with the muscarinic receptors inhibits the coupled adenylate cyclase, decreasing the conversion of adenosine triphosphate (ATP) to adenosine monophosphate (cAMP). This ultimately reduces the intracellular calcium levels, prolonging repolarization and refractory period and thus decreasing cardiac automaticity. Of note, this mechanism is similar to beta-blockade, vagal maneuvers, and adenosine, which have been used as antiarrhythmic treatments [38–43].

In addition to its direct parasympathetic mechanism of action, dexmedetomidine binds to alpha-2 adrenoreceptors located on presynaptic sympathetic neurons. This provides negative feedback to the presynaptic neuron, preventing the synaptic vesicles from releasing norepinephrine [42]. In healthy human subjects, the infusion of dexmedetomidine decreases the plasma level of norepinephrine and epinephrine [44]. High catecholamine burden and subsequent cardiac beta-adrenergic receptors activation is a major mediator of ventricular and supraventricular arrhythmias [13].

More recently, Yang et al. demonstrated that dexmedetomidine acts on sodium and calcium channels, dose-dependently inhibiting the frequency of spontaneous ventricular-like action potential in human induced pluripotent stem cell-derived cardiomyocytes (hiPSC-CMs). This novel mechanism proposes that dexmedetomidine may act directly on myocardial cells, bypassing the alpha-adrenoceptor [45].

Research on the antiarrhythmic effects of dexmedetomidine for clinical practice has been limited to animal models and pediatric patients. Early animal studies showed that dexmedetomidine increased the arrhythmogenic threshold of epinephrine in a dose-dependent manner in halothane-anesthetized dogs [35]. Parent et al. reported a case of terminating recurrent ventricular tachycardia with dexmedetomidine infusion in a 12-year-old patient [13]. Chrysostomou et al. described successful treatment of supraventricular, junctional, and ventricular tachyarrhythmias with dexmedetomidine in a pediatric population [34, 46]. Tobias et al. provided a literature review highlighting the decreased incidence of postoperative ventricular and supraventricular tachyarrhythmias with dexmedetomidine use during pediatric cardiac surgery [42].

Our meta-analysis is the most extensive study to date to examine the incidences of VT, VF, and overall VA in ICU adult patients. We demonstrate that dexmedetomidine reduces the risk of VA by 65% compared to control. Furthermore, dexmedetomidine minimizes the risk of VT by 75%, and this reduction of VT risk is what primarily drives the reduction of VA incidence with dexmedetomidine. VF risk, notably, is not affected by dexmedetomidine. Patients presenting with VF or pulseless VT prompts the initiation of the Advanced Cardiovascular Life Support (ACLS) algorithm, while patients presenting with VT with pulse are treated with sedation, beta blockade, and antiarrhythmics. Differences in presenting rhythm, patient stability, and treatment pathways may explain the difference in outcomes between VT and VF. More research is needed to explore the differences in outcomes between VT with and without a pulse.

Our meta-analysis provides the following distinctions and updates compared to the prior meta-analyses on the treatment of cardiac arrhythmia with dexmedetomidine [10, 28, 29, 47]. First, our paper has compiled the most significant number of RCTs and patient sample size. Compared to the most recent meta-analyses by Liu [10] and Ling et al. [47] which both included 5 RCTs, our meta-analysis included one additional RCT. Second, we report on the overall VA incidence as well as the subgroup analysis of VT and VF incidences. Third, unlike previously published meta-analyses, our study design highlights ventricular arrhythmias as the primary endpoint. Fourth, our search strategy was purposefully extensive to cover all adult ICU patients. Prior meta-analyses have focused explicitly on postcardiac surgery patients. For example, the search criteria employed by Liu et al. concentrated on the incidence of atrial fibrillation in adult patients undergoing cardiac surgery for their primary outcome and reported VT and VF as secondary outcomes [10]. Geng et al. focused on the safety and efficacy of dexmedetomidine following cardiac surgery, reporting on results such as duration of mechanical ventilation, bradycardia, hypotension, and delirium, along with VT [28]. Lin et al. reported on the use of dexmedetomidine for postoperative sedation in elective cardiac surgery on the duration of mechanical ventilation, delirium, hyperglycemia, and VT [29]. Ling et al. analyzed outcomes of cardiac arrhythmias associated with dexmedetomidine postcardiac surgery and found a lower incidence of VA [47].

Our literature review highlights the need for more prospective RCTs studying the antiarrhythmic properties of dexmedetomidine in the general adult ICU population. The focus of VA in the perioperative state is unsurprising, given the high risk of ventricular and supraventricular tachyarrhythmias seen after the catecholamine surge associated with cardiac surgery and cardiopulmonary bypass [42]. Still, this review highlights the dearth of RCTs examining the use of dexmedetomidine in instances of ventricular arrhythmias with other hypercatecholaminergic states such as sepsis, major organ failure, neurological injury, trauma, and noncardiac surgeries. Studies are needed to assess if the antiarrhythmic properties of dexmedetomidine can be extrapolated to all adult ICU patients.

### 4.2. Hemodynamic Stability

Bradycardia and hypotension are the common adverse events reported with dexmedetomidine [16, 30]. Some studies have suggested a higher incidence of hypertension [48], hypotension [16, 49–51], and bradycardia [51, 52] during loading or rapid dose escalation of dexmedetomidine. Our meta-analysis demonstrates an elevated risk of bradycardia with dexmedetomidine but no difference in risk of hypotension. One potential explanation is that the degree of bradycardia was not severe enough to cause hypotension, and the circulatory system was able to compensate for the decreased cardiac output. Second, aside from the indirect parasympathomimetic and sympatholytic mechanisms, dexmedetomidine may directly inhibit cardiac myocytes by affecting sodium and calcium channels. In hiPSC-CMs, dexmedetomidine decreases the frequency and prolongs the duration of action potentials [45]. Notably, loading doses of dexmedetomidine were employed in 2 out of the 4 studies that reported outcomes on bradycardia included in our meta-analysis [24, 25]. Using a low loading rate or even forgoing the loading dose and, therefore, may reduce hemodynamic instability. In addition, specific patient characteristics such as preexisting low blood pressure, history of coronary artery disease, and higher acuity have also been identified as independent risk factors for dexmedetomidine-associated hypotension [49]. More research is needed to identify patient characteristics that may signify higher risks for dexmedetomidine-associated bradycardia.

### 4.3. Mortality

Dexmedetomidine does not have the adverse effect of respiratory depression that is seen in traditional ICU sedatives. Because dexmedetomidine's highly selective alpha-2 agonism does not interact with the gamma-aminobutyric acid (GABA) receptors [14, 29], its analgesic properties are considered opioid-sparing [15]. The ICU liberation guidelines recommend considering dexmedetomidine before benzodiazepines because of shorter ventilation time, which could shorten mechanical ventilation time and ICU days.

Current literature does not show mortality benefit with dexmedetomidine [53–57]. Of note, these studies did not focus on patients with arrhythmias, but rather the general ICU population. Our meta-analysis also shows no significant mortality difference between dexmedetomidine and the control group. There are several caveats to these findings. First, the mortality endpoint varied among the included studies, ranging from ICU mortality to in-hospital mortality. Second, there was significant heterogeneity regarding the mortality data for these studies, as indicated by the I^2^ of 71%. Thus, our mortality results should be interpreted with caution.

### 4.4. Limitations

There are several limitations with this meta-analysis. First, all the included studies involved perioperative cardiac surgery patients. Thus, more research is necessary to assess if these results may be extrapolated to noncardiac surgery patients and the general ICU population. Second, the assessment of sedation was inconsistent across studies with some studies not reporting a sedation goal, while others using the RSS or MAAS level. Third, the control drugs used by the included studies were heterogeneous, comprising placebo, propofol, or morphine. Further comparisons of dexmedetomidine with other traditional ICU sedatives such as benzodiazepines and fentanyl are necessary.

## 5. Conclusion

Sustained ventricular tachycardia and ventricular fibrillation portend a high morbidity and mortality [1–3]. This current meta-analysis suggests that dexmedetomidine is associated with decreased ventricular tachycardia (VT) incidence, but not ventricular fibrillation (VF). Further prospective research is necessary to clarify dexmedetomidine's antiarrhythmic and sedative properties and its potential effect on patient outcomes.

## Figures and Tables

**Table 1:** Study design of included randomized controlled trials.

Author (publication year) (citation)	Study design	# of patients	Sedation goal	Dex dose	Dex intervention time	Control
Jaionen et al. [25]	Double blind, parallel-group, randomized controlled trial	80	Unspecified	Loading dose 50 ng/kg/min for 30 mins and then maintained at 7 ng/kg/min	30 mins before initiation of surgical anesthesia—end of surgery	Saline placebo

Corbett et al. [23]	Prospective, randomized study	89	RSS of 5 for the first 2 h postoperative, followed by a score of 3 to 4 during intubation	Loading dose of 1 *μ*g/kg induction and then maintained by 0.4 *μ*g/kg/h	ICU admission—1 hr postextubation	Propofol: 0.2 to 0.7 *μ*g/kg/h

Shehabi et al. [20]	Randomized, double-blinded controlled trial	299	MAAS 2–4	0.1 to 0.7 *μ*g/kg/h	ICU admission—extubation/leaving the ICU/48 h maximum	Morphine: 10 to 70 *μ*g/kg/h

Herr et al. [24]	Randomized, open label study	295	RSS ≥3 during assisted ventilation and ≥2 after extubation	Loading dose of 1.0 *μ*g/kg and then maintained by 0.2 to 0.7 *μ*g/kg/h	Sternal closure—24 h in the ICU	Propofol: unspecified dose

Ren et al. [22]	Randomized controlled trial	162	Unspecified	0.2–0.5 *μ*g/kg/h	Following the first vascular anastomosis grafting—12 h in the CICU	Saline placebo

Soltani et al.[27]	Randomized, blinded clinical trial	76	BIS 40–60	0.5 *μ*g/kg/h	Surgical induction—transfer to CICU	Saline placebo

Dex, dexmedetomidine; MAAS, motor activity assessment scale; RSS, ramsay sedation score; BIS, bispectral index; CPB, cardiopulmonary bypass; CICU, cardiac intensive care unit; ICU, intensive care unit.

**Table 2:** Patient characteristics of included randomized controlled trials.

Author (publication year)	Age	Male (%)	Weight (kg)	HTN (%)	Prior MI (%)	Duration of surgery (min)
Jalonen (1997)	55.4	83.8	80.4	—	53.8	182.5
Corbett (2005)	62.7	82.0	88.7	—	—	195.7
Shehabi (2009)	71.3	75.3	—	85.1	36.9	—
Herr (2003)	62.1	—	84.6	—	—	—
Ren (2013)	58.0	32.5	—	80.0	—	—
Soltani (2017)	59.9	40.8	72.9	68.4	1.3	297.0

HTN, hypertension; MI, myocardial infarction.

## Data Availability

All data used in this meta-analysis may be found in online journals or by search databases.

## Supplementary Materials

Supplementary Materials Search strategy.
