# Supplementary material for: Dexmedetomidine Reduces Incidences of Ventricular Arrhythmias in Adult Patients: A Meta-Analysis
Source: Cardiol Res Pract. 2022 Jun 1;2022:5158362. doi: 10.1155/2022/5158362 (PMC9177331; doi:10.1155/2022/5158362)
Supplement: Supplementary Materials — Search strategy. [file 5158362.f1.docx]

**Supplementary Material: Search Strategy**

**OVID**

Database(s): **Ovid MEDLINE(R) 1946 to Present and Epub Ahead of Print, In-Process & Other Non-Indexed Citations and Ovid MEDLINE(R) Daily, EBM Reviews - Cochrane Central Register of Controlled Trials**October 2020**, EBM Reviews - Cochrane Database of Systematic Reviews**2005 to November 19, 2020**, Embase**1974 to 2020 November 24
Search Strategy:

| **#** | **Searches** |
| --- | --- |
| 1 | (dexmedetomidine or precedex).ti,ab,hw,kw. |
| 2 | dexmedetomidine/ |
| 3 | 1 or 2 |
| 4 | Arrhythmias, Cardiac/ or exp *Arrhythmias, Cardiac/ |
| 5 | "Tachycardia, Ventricular"/ or exp heart ventricle tachycardia/ |
| 6 | (arrhythm* or anti-arrhythm* or antiarrhythm* or tachyarrhythmia* or tachycardia* or bradycardia).ti,ab. |
| 7 | or/4-6 |
| 8 | exp "Intensive Care Units"/ |
| 9 | ("intensive care" or ICU).ti,ab,hw,kw. |
| 10 | Critical Care/ or Coronary Care Units/ |
| 11 | ("intensive cardiac unit" or MICU or "medical intensive care unit" or CCU or "coronary care unit" or CICU or " cardiac intensive care unit" or "critical care unit").ti,ab,hw,kw. |
| 12 | or/8-11 |
| 13 | 3 and 7 and 12 |
| 14 | ((randomized adj3 study) or (randomized adj3 trial) or (randomised adj3 study) or (randomised adj3 trial) or "pragmatic clinical trial" or (doubl* adj1 blind*) or (doubl* adj1 mask*) or (singl* adj blind*) or (singl* adj mask*) or (tripl* adj blind*) or (tripl* adj mask*) or (trebl* adj blind*) or (trebl* adj mask*) or "latin square" or placebo* or nocebo* or random*).mp,pt. |
| 15 | exp Randomized Controlled Trial/ |
| 16 | exp triple blind procedure/ |
| 17 | exp Double-Blind Method/ |
| 18 | exp Single-Blind Method/ |
| 19 | exp latin square design/ |
| 20 | exp Placebos/ |
| 21 | exp Placebo Effect/ |
| 22 | or/14-21 |
| 23 | 13 and 22 |
| 24 | limit 23 to english language [Limit not valid in CDSR; records were retained] |
| 25 | (conference abstract or conference review or editorial or erratum or note or addresses or autobiography or bibliography or biography or blogs or comment or dictionary or directory or interactive tutorial or interview or lectures or legal cases or legislation or news or newspaper article or patient education handout or periodical index or portraits or published erratum or video-audio media or webcasts).mp. or conference abstract.st. |
| 26 | 24 not 25 |
| 27 | 26 not ((exp animals/ or exp nonhuman/) not exp humans/) |
| 28 | remove duplicates from 27 |

**SCOPUS**

| 1 | (dexmedetomidine or precedex) |
| --- | --- |
| 2 | (arrhythm* or anti-arrhythm* or antiarrhythm* or tachyarrhythmia* or tachycardia* or bradycardia) |
| 3 | ("intensive care" or ICU or "intensive cardiac unit" or MICU or "medical intensive care unit" or CCU or "coronary care unit" or CICU or " cardiac intensive care unit" or "critical care unit") |
| 4 | 1 and 2 and 3 |
| 5 | TITLE-ABS-KEY ( ( randomized W/3 study ) OR ( randomized W/3 trial ) OR ( randomised W/3 study ) OR ( randomised W/3 trial ) OR "pragmatic clinical trial" OR ( doubl* W/1 blind* ) OR ( doubl* W/1 mask* ) OR ( singl* W/1 blind* ) OR ( singl* W/1 mask* ) OR ( tripl* W/1 blind* ) OR ( tripl* W/1 mask* ) OR ( trebl* W/1 blind* ) OR ( trebl* W/1 mask* ) OR "latin square" OR placebo* OR nocebo* OR random* ) |
| 6 | TITLE-ABS-KEY ( ( clinical OR intervention* OR evaluation OR validation OR cohort OR comparative OR prospective* OR retrospective OR "cross section*" OR random* ) W/4 ( trial* OR stud* ) ) |
| 7 | 5 or 6 |
| 8 | INDEX(embase) OR INDEX(medline) OR PMID(0* OR 1* OR 2* OR 3* OR 4* OR 5* OR 6* OR 7* OR 8* OR 9*) |
| 9 | 7 not 8 |
| 10 | DOCTYPE(ed) OR DOCTYPE(bk) OR DOCTYPE(er) OR DOCTYPE(no) OR DOCTYPE(sh) OR DOCTYPE(ch) |
| 11 | 9 not 10 |
| 12 | LANGUAGE(english) |
| 13 | 11 and 12 |
| 14 | ( TITLE-ABS-KEY ( ( alpaca OR alpacas OR amphibian OR amphibians OR animal OR animals OR antelope OR armadillo OR armadillos OR avian OR baboon OR baboons OR beagle OR beagles OR bee OR bees OR bird OR birds OR bison OR bovine OR buffalo OR buffaloes OR buffalos OR "c elegans" OR "Caenorhabditis elegans" OR camel OR camels OR canine OR canines OR carp OR cats OR cattle OR chick OR chicken OR chickens OR chicks OR chimp OR chimpanze OR chimpanzees OR chimps OR cow OR cows OR "D melanogaster" OR "dairy calf" OR "dairy calves" OR deer OR dog OR dogs OR donkey OR donkeys OR drosophila OR "Drosophila melanogaster" OR duck OR duckling OR ducklings OR ducks OR equid OR equids OR equine OR equines OR feline OR felines OR ferret OR ferrets OR finch OR finches OR fish OR flatworm OR flatworms OR fox OR foxes OR frog OR frogs OR "fruit flies" OR "fruit fly" OR "G mellonella" OR "Galleria mellonella" OR geese OR gerbil OR gerbils OR goat OR goats OR goose OR gorilla OR gorillas OR hamster OR hamsters OR hare OR hares OR heifer OR heifers OR horse OR horses OR insect OR insects OR jellyfish OR kangaroo OR kangaroos OR kitten OR kittens OR lagomorph OR lagomorphs OR lamb OR lambs OR llama OR llamas OR macaque OR macaques OR macaw OR macaws OR marmoset OR marmosets OR mice OR minipig OR minipigs OR mink OR minks OR monkey OR monkeys OR mouse OR mule OR mules OR nematode OR nematodes OR octopus OR octopuses OR orangutan OR "orang-utan" OR orangutans OR "orang-utans" OR oxen OR parrot OR parrots OR pig OR pigeon OR pigeons OR piglet OR piglets OR pigs OR porcine OR primate OR primates OR quail OR rabbit OR rabbits OR rat OR rats OR reptile OR reptiles OR rodent OR rodents OR ruminant OR ruminants OR salmon OR sheep OR shrimp OR slug OR slugs OR swine OR tamarin OR tamarins OR toad OR toads OR trout OR urchin OR urchins OR vole OR voles OR waxworm OR waxworms OR worm OR worms OR xenopus OR "zebra fish" OR zebrafish ) AND NOT ( human OR humans OR patient OR patients ) ) ) |
| 15 | 13 not 14 |
